# Genome resources—A chromosome-level genome assembly for the long-nosed leopard lizard, *Gambelia wislizenii*, the first reference genome for the lizard family Crotaphytidae

**DOI:** 10.1093/jhered/esaf053

**Published:** 2025-07-31

**Authors:** Jimmy A McGuire, Jonathan Q Richmond, Merly Escalona, Mohan P A Marimuthu, Oanh Nguyen, Samuel Sacco, Eric Beraut, Erin Toffelmier, Robert D Cooper, Michael Westphal, Robert N Fisher, Ian J Wang, H Bradley Shaffer

**Affiliations:** Museum of Vertebrate Zoology, University of California, Berkeley, CA 94720, United States; Department of Integrative Biology, University of California, Berkeley, CA 94720, United States; U.S. Geological Survey, 4165 Spruance Rd. Suite 200, San Diego, CA 92101, United States; Department of Biomolecular Engineering, University of California, Santa Cruz, CA 95064, United States; DNA Technologies and Expression Analysis Core Laboratory, Genome Center, University of California, Davis, CA 95616, United States; DNA Technologies and Expression Analysis Core Laboratory, Genome Center, University of California, Davis, CA 95616, United States; Department of Ecology and Evolutionary Biology, University of California, Santa Cruz, CA 95064, United States; Department of Ecology and Evolutionary Biology, University of California, Santa Cruz, CA 95064, United States; Department of Ecology & Evolutionary Biology, University of California, Los Angeles, CA 90095-7239, United States; La Kretz Center for California Conservation Science, Institute of the Environment and Sustainability, University of California, Los Angeles, CA 90095-7239, United States; Department of Ecology & Evolutionary Biology, University of California, Los Angeles, CA 90095-7239, United States; La Kretz Center for California Conservation Science, Institute of the Environment and Sustainability, University of California, Los Angeles, CA 90095-7239, United States; Central Coast Field Office, United States Bureau of Land Management, 940 2nd Ave., Marina, CA 93933, United States; U.S. Geological Survey, 4165 Spruance Rd. Suite 200, San Diego, CA 92101, United States; Museum of Vertebrate Zoology, University of California, Berkeley, CA 94720, United States; Department of Environmental Science, Policy, and Management, University of California, Berkeley, CA 94720, United States; Department of Ecology & Evolutionary Biology, University of California, Los Angeles, CA 90095-7239, United States; La Kretz Center for California Conservation Science, Institute of the Environment and Sustainability, University of California, Los Angeles, CA 90095-7239, United States

**Keywords:** California conservation genomics project, conservation genetics, Crotaphytidae, endangered species, genome assembly

## Abstract

We report on an annotated chromosome-level genome assembly for the long-nosed leopard lizard, *Gambelia wislizenii*, as part of the California Conservation Genomics Project (CCGP). All 17 species of reptiles, including two turtles, seven lizards, and seven snakes targeted for reference genome sequencing by the CCGP are now complete and posted on NCBI, and this article is the third of seven CCGP lizard release papers to be published. It is also the first species of the family Crotaphytidae to have a released reference genome*.* Following the CCGP pipeline, the *G. wislizenii* genome was produced using Pacific Biosciences HiFi long reads and Omni-C proximity ligation data. The de novo assembly includes 69 scaffolds and has a total length of ~ 2.47 Gb, a scaffold N50 length of 380.1 Mb, and a BUSCO completeness score of 97.4% based on the tetrapod gene set. We improved the annotation of the genome using transcriptome sequencing (seven tissue types), identifying 23,279 genes, with BUSCO completeness of 98.9%. This reference genome, when combined with CCGP’s on-going state-wide resequencing efforts for the three species of *Gambelia* in California, including the federally endangered blunt-nosed leopard lizard (*Gambelia sila*), and Cope’s leopard lizard (*Gambelia copei*), will be a powerful tool enabling researchers to characterize hybridization dynamics between *Gambelia* species, document the remaining diversity within *G. sila*, and explore the genetic underpinnings of key traits that vary between the three *Gambelia* species, such as territoriality, sexual size dimorphism, presence versus absence of male breeding coloration, and skull morphologies.

Leopard lizards of the genus *Gambelia* are widespread denizens of arid landscapes across much of western North America. These relatively large lizards are well-known for their predatory habits, preying on a diversity of small vertebrates and arthropods (Tanner and Krogh 1974; Parker and Pianka 1976; Tollestrup 1983). The genus contains three species, all of which occur in California. The long-nosed leopard lizard, *Gambelia wislizenii*, is the most broadly distributed, with a range including Sonoran, Mohave, Great Basin, and Chihuahuan desert habitats. In contrast, the state and federally endangered blunt-nose leopard lizard, *Gambelia sila*, is endemic to California’s San Joaquin Desert (see Germano et al. 2011; Stewart et al. 2019), and Cope’s leopard lizard, *Gambelia copei*, is found throughout much of the Baja California Peninsula, barely entering California at a few documented sites adjacent to the Mexican border (McGuire 1996; Mahrdt et al. 2010). Because a primary aim of the California Conservation Genomics Project (CCGP) is to document landscape-scale genetic diversity across the state for a diverse set of taxa (Fiedler et al. 2022; Shaffer et al. 2022), a high-quality reference genome for any one species can be paired with re-sequenced genomes for each of the three *Gambelia* species, providing landscape genomic data spanning most of the arid landscapes of the state.

The three *Gambelia* species share numerous phenotypic and ecological traits. All three species are relatively large and share the unusual habit (for a lizard) of consuming vertebrates, especially other lizards, as part of their diets (although *G. sila* is thought to prey on vertebrates less frequently than the larger *G. wislizenii* and *G. copei*; Montanucci 1965; Germano et al. 1994, 2007). Like other crotaphytids, gravid females of all three species display conspicuous red or orange coloration that develops shortly before ovulation and dissipates post-parturition. However, *Gambelia* species also exhibit important interspecific differences. In particular, *G. wislizenii* and *G. copei* exhibit female-biased sexual size dimorphism apparently tied to their loss of territoriality, whereas the territorial *G. sila* exhibits male-biased size dimorphism that is typically associated with territorial defense (McCoy 1967; Montanucci 1970; McGuire 1996). Perhaps as a result, the non-territorial *G. copei* and *G. wislizenii* lack male breeding coloration, whereas *G. sila* males exhibit vibrant pink to rusty red coloration on the head and trunk during the breeding period (Montanucci 1965). *Gambelia wislizenii* and *G. copei* also differ from *G. sila* in having relatively longer snouts than the aptly named blunt-nosed leopard lizard (McGuire 1996; Lappin and Swinney 1999). All three species are closely related and were previously considered to be conspecific, and gene flow has been documented between *G. wislizenii* and *G. sila* (Montanucci 1970; Richmond et al. 2017) and may occur between *G. wislizenii* and *G. copei* where they meet in Paseo de San Matias in northern Baja California, Mexico (McGuire 1996).

Here, we present the first annotated assembled reference genome for *G. wislizenii*, generated as part of the CCGP (Beninde et al. 2022; Fiedler et al. 2022; Shaffer et al. 2022; Toffelmier et al. 2022). The *G. wislizenii* genome assembly joins 39 other genomes representing the cosmopolitan iguanian lizard clade, including two other CCGP species: Blainville’s horned lizard, *Phrynosoma blainvillii* (Richmond et al. 2023), and the western fence lizard, *Sceloporus occidentalis* (Bishop et al. 2023). It is the first reference genome for the iguanian family Crotaphytidae. Combined with the landscape genomics approach of the CCGP, this genome will be a powerful tool enabling researchers to characterize hybridization dynamics between *G. wislizenii* and *G. sila* and possibly *G. wislizenii* and *G. copei*, document the remaining diversity within *G. sila* relative to its more widespread congeners, and explore the genetic underpinnings of key traits that vary between the three *Gambelia* species.

## Methods

### Biological materials

**Fig. 1:**
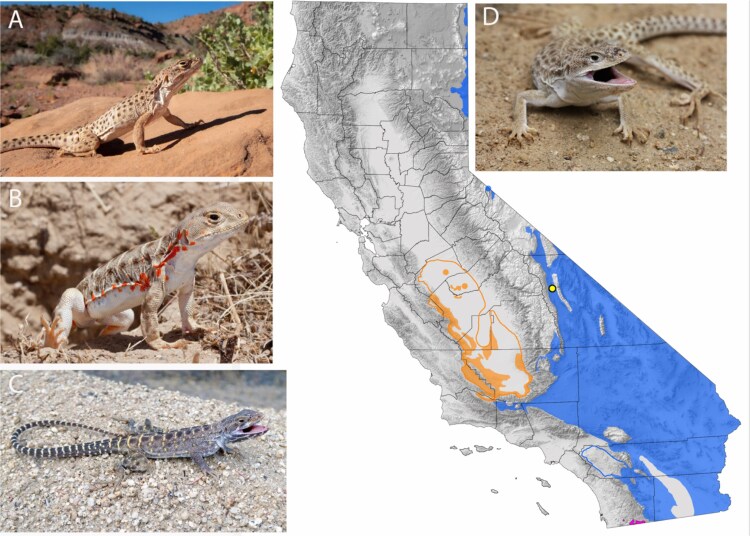
Distribution of *Gambelia wislizenii* (blue), *G. sila* (orange), and *G. copei* (magenta) in California modified from Hansen and Shedd (2025). The yellow dot indicates the collection site for the genome voucher specimen (mouth of French Canyon, Inyo Mountains, Inyo County (36.6565016, −118.0111543). A) Adult male *G. wsislizenii* basking in San Juan Co., Utah (B. Oldfield); B) gravid female *G. sila* from the Carrizo Plain National Monument, San Luis Obispo County illustrating gravid coloration (B. Bouton, CC BY-NC-SA); C) *G. copei* from Campo, San Diego County (JQ Richmond); and D) *G. wislizenii* reference genome voucher sample from the mouth of French Canyon, Inyo Mountains, Inyo County (H.B. Shaffer).

We captured, euthanized, and flash froze multiple tissues (blood, brain, intestine, liver, lung, muscle, and ovary) from a *G. wislizenii* collected from the mouth of French Canyon, Inyo Mountains, Inyo County, California (Fig. 1: 36.6565016, −118.0111543) on 23 April 2021 (CDFW entity permit no. SC-838). The specimen (female, field number HBS 135999, MVZ:Herp:300824) was euthanized, tissues were harvested, and the voucher was preserved in formalin and accessioned at the Museum of Vertebrate Zoology, University of California at Berkeley for further research use.

### High-molecular-weight genomic DNA isolation

We extracted high molecular weight (HMW) genomic DNA (gDNA) from 30 mg of liver tissue using the Nanobind Tissue Big DNA kit as per the manufacturer’s instructions (Pacific BioSciences—PacBio, Menlo Park, CA). We estimated DNA purity based on absorbance ratios (260/280 = 1.83 and 260/230 = 2.11) from a NanoDrop ND-1000 spectrophotometer and quantified the final DNA yield (11.2 μg) using Qubit 2.0 Fluorometer (Thermo Fisher Scientific, MA). Eighty-eight percent of the DNA fragments were 50 kb or longer based on the sizing of HMW gDNA on the Femto Pulse system (Agilent, Santa Clara, CA).

### HiFi library preparation and sequencing

The HiFi SMRTbell library was constructed using the SMRTbell Express Template Prep Kit v2.0 (PacBio, Cat. #100-938-900) according to the manufacturer’s instructions. HMW gDNA was sheared to a target DNA size distribution between 15 and 18 kb using Diagenode’s Megaruptor 3 system (Diagenode, Belgium; Cat. B06010003). The sheared gDNA was concentrated using 0.45X of AMPure PB beads (PacBio, Cat. #100-265-900) for the removal of single-strand overhangs at 37°C for 15 min. This was followed by enzymatic DNA damage repair at 37°C for 30 min, end repair and A-tailing at 20°C for 10 min and 65°C for 30 min, and the ligation of overhang adapters v3 at 20°C for 60 min. The SMRTbell library was purified and concentrated with 1X AMPure PB beads for nuclease treatment at 37°C for 30 min and size selection using the PippinHT system (Sage Science, Beverly, MA; Cat #HPE7510) to collect fragments greater than 7 to 9 kb. The 15 to 20 kb average HiFi SMRTbell library was sequenced at UC Davis DNA Technologies Core (Davis, CA) using four 8 M SMRT cells, Sequel II sequencing chemistry 2.0, and 30-h movies each on a PacBio Sequel II sequencer.

### Omni-C library preparation

The Omni-C library was prepared using the Dovetail Omni-C Kit (Dovetail Genomics, CA) according to the manufacturer’s protocol with slight modifications. First, specimen tissue (liver, ID: HBS 135999) was thoroughly ground with a mortar and pestle while cooled with liquid nitrogen. Subsequently, chromatin was fixed in place in the nucleus. The suspended chromatin solution was then passed through 100 and 40 μm cell strainers to remove large debris. Fixed chromatin was digested under various conditions of DNase I until a suitable fragment length distribution of DNA molecules was obtained. Chromatin ends were repaired and ligated to a biotinylated bridge adapter followed by proximity ligation of adapter containing ends. After proximity ligation, crosslinks were reversed and the DNA purified from proteins. Purified DNA was treated to remove biotin that was not internal to ligated fragments. An NGS library was generated using an NEB Ultra II DNA Library Prep Kit (New England Biolabs, Ipswich, MA) with an Illumina compatible y-adaptor. Biotin-containing fragments were then captured using streptavidin beads. The post capture product was split into two replicates prior to polymerase chain reaction enrichment to preserve library complexity with each replicate receiving unique dual indices. The library was sequenced at Vincent J. Coates Genomics Sequencing Lab (Berkeley, CA) on an Illumina NovaSeq platform (Illumina, CA) to generate approximately 100 million 2 × 150 bp read pairs per GB genome size.

### Transcriptome RNA extraction, library preparation, and sequencing

Total RNA was extracted from seven separate tissues from the same individual (blood, brain, intestine, liver, lung, muscle, and ovary) using a Qiagen RNeasy Mini Kit (Qiagen, Netherlands) according to the manufacturer’s protocol. RNA libraries were then prepared using the KAPA mRNA HyperPrep Kit (Roche, Switzerland) according to the manufacturer’s protocol. Libraries were sequenced using 150 bp paired end reads on an Illumina NovaSeq 6000 platform (Illumina, San Diego, CA), to generate approximately 50 M reads per library.

### Nuclear genome assembly

We assembled the genome of a female *G. wislizenii* following the CCGP assembly pipeline Version 5.0, as outlined in Table 1 where we list the tools and non-default parameters used in the assembly process. We removed the remnant adapter sequences from the PacBio HiFi dataset using HiFiAdapterFilt (Sim et al. 2022) and generated an initial diploid phased assembly using HiFiasm (Cheng et al. 2022) in “HiC” mode with the filtered PacBio HiFi reads and the Omni-C short-reads. This generates two assemblies, one per haplotype. We then aligned the Omni-C data to both assemblies separately following the Arima Genomics Mapping Pipeline (https://github.com/ArimaGenomics/mapping_pipeline) and then scaffolded both assemblies with SALSA (Ghurye et al. 2017; Ghurye et al. 2019).

**Table 1:** Assembly pipeline and software used.

**Assembly step**	**Software and any non-default options**	**Version**
**Initial assembly**		
Filtering PacBio HiFi adapters	HiFiAdapterFilt	Commit 64d1c7b
K-mer counting	Meryl (*k* = 21)	1
Estimation of genome size and heterozygosity	GenomeScope	2
De novo assembly (contiging)	HiFiasm (Hi-C Mode, -primary, output hic.hap1.p_ctg, hic.hap2.p_ctg)	0.16.1-r375
**Scaffolding**		
Omni-C data alignment	Arima Genomics Mapping Pipeline	Commit 2e74ea4
Arima Genomics Mapping Pipeline (AGMP)	BWA-MEM	0.7.17-r1188
	samtools	1.11
	filter_five_end.pl (AGMP)	Commit 2e74ea4
	two_read_bam_combiner.pl ((AGMP))	Commit 2e74ea4
	picard	2.27.5
Omni-C scaffolding	SALSA (-DNASE, -i 20, -p yes)	2
**Omni-C contact map generation**		
Short-read alignment	BWA-MEM (-5SP)	0.7.17-r1188
SAM/BAM processing	samtools	1.11
SAM/BAM filtering	pairtools	0.3.0
Pairs indexing	pairix	0.3.7
Matrix generation	cooler	0.8.10
Matrix balancing	hicExplorer (hicCorrectmatrix correct --filterThreshold -2 4)	3.6
Contact map visualization	HiGlass	2.1.11
	PretextMap	0.1.4
	PretextView	0.1.5
	PretextSnapshot	0.0.3
Manual curation tools	Rapid curation pipeline (Wellcome Trust Sanger Institute, Genome Reference Informatics Team)	Commit 7acf220c
**Genome quality assessment**		
Basic assembly metrics	QUAST (--est-ref-size)	5.0.2
Assembly completeness	BUSCO (-m geno, -l tetrapoda)	5.0.0
	Merqury	29 January 2020
**Contamination screening**		
Local alignment tool	BLAST+ (-db nt, -outfmt ‘6 qseqid staxids bitscore std’, -max_target_seqs 1, -max_hsps 1, -evalue 1e-25)	2.15
General contamination screening	BlobToolKit (HiFi coverage, BUSCO = tetrapoda, NCBI Taxa ID = 43593)	2.3.3
**Mitochondrial assembly**		
Mitochondrial genome assembly	MitoHiFi (-r, -p 90, -o 1) Reference: *Gambelia wislizenii* (NCBI:NC_012831.1)	2.2
MitoHiFi: Mitochondrial genome annotation	MitoFinder (MitoHiFi pipeline parameters)	1.4
**Genome annotation**		
Genome annotation	NCBI Eukaryotic Genome Annotation Pipeline (“https://github.com/ncbi/egapx”)	0.3.2-alpha
Local alignment of transcripts and proteins	BLAST	
HMM-based gene prediction	NCBI Gnommon pipeline	
Alingment of RNA-seq reads	STAR	
Gene identification	BUSCO	

The assemblies for both haplotypes were manually curated by iteratively generating and analyzing their corresponding Omni-C contact maps. To generate the contact maps we aligned the Omni-C data with BWA-MEM (Li 2013), identified ligation junctions, and generated Omni-C pairs (Lee et al. 2022) using pairtools (Open2C et al. 2024). Then, we generated multi-resolution Omni-C matrices with cooler (Abdennur and Mirny 2020) and balanced them with hicExplorer (Ramírez et al. 2018). We used HiGlass (Kerpedjiev et al. 2018) and the PretextSuite (https://github.com/wtsi-hpag/PretextView;  https://github.com/wtsi-hpag/PretextMap;  https://github.com/wtsi-hpag/PretextSnapshot) to visualize the contact maps. We identified misassemblies and misjoins in these contact maps and modified the assemblies using the Rapid Curation pipeline from the Wellcome Trust Sanger Institute, Genome Reference Informatics Team (https://gitlab.com/wtsi-grit/rapid-curation). Some of the remaining gaps (joins generated during scaffolding and/or curation) were closed using the PacBio HiFi reads and YAGCloser (https://github.com/merlyescalona/yagcloser). We checked for contamination using the BlobToolKit Framework (Challis et al. 2020).

### Mitochondrial genome assembly

We assembled the mitochondrial genome from the PacBio HiFi reads using the reference-guided pipeline MitoHiFi (Allio et al. 2020; Uliano-Silva et al. 2023). The mitochondrial sequence of a *G. wislizenii* (NCBI:NC_012831.1) was used as the starting sequence. After completion of the nuclear genome, we searched for matches of the resulting mitochondrial assembly sequence in the nuclear genome assembly using BLAST+ (Camacho et al. 2009) and filtered out contigs and scaffolds from the nuclear genome with a percentage of sequence identity > 99% and size smaller than the mitochondrial assembly sequence.

### Genome size estimation and quality assembly assessment

We generated k-mer counts from the PacBio HiFi reads using meryl (https://github.com/marbl/meryl). The k-mer counts were then used in GenomeScope2.0 (Ranallo-Benavidez et al. 2020) to estimate genome features including genome size, heterozygosity, and repeat content. To obtain general contiguity metrics, we ran QUAST (Gurevich et al. 2013). To evaluate genome quality and functional completeness, we used BUSCO (Manni et al. 2021) with the Tetrapoda ortholog database (tetrapoda_odb10) which contains 5,286 genes. Assessment of base level accuracy (QV) and k-mer completeness was performed using the previously generated meryl database and merqury (Rhie et al. 2020). We further estimated genome assembly accuracy via BUSCO gene set frameshift analysis using the pipeline described by Korlach et al. (2017). Measurements of the size of the phased blocks is based on the size of the contigs generated by HiFiasm on HiC mode. We follow the quality metric nomenclature established by Rhie et al. (2021), with the genome quality code x.y.P.Q.C, where, x = log10[contig NG50]; y = log10[scaffold NG50]; P = log10 [phased block NG50]; Q = Phred base accuracy QV (quality value); and C = % genome represented by the first “*n*” scaffolds, following a karyotype of 2*n* = 36 for this species, estimated as a mode from ancestral species number of chromosomes [Genome on a Tree—GoaT; tax_name (*G. wislizenii*); Porter et al. 1994; Challis et al. 2023]. Quality metrics for the notation were calculated on the haplotype one assembly.

**Table 2:** Sequencing and assembly statistics and accession numbers.

Bio projects and vouchers	CCGP NCBI BioProject			PRJNA720569			
	Genus NCBI BioProject			PRJNA765827			
	Species NCBI BioProject			PRJNA986198			
	Transcriptome NCBI BioProject			PRJNA1011925			
	NCBI Assembly BioSample			SAMN35845454			
	NCBI Transcriptome BioSamples			SAMN40863703, SAMN40863704, SAMN40863705, SAMN40863706, SAMN40863707, SAMN40863708, SAMN40863709			
	Specimen identification			HBS 135999			
	NCBI Genome accessions			**Haplotype 1**		**Haplotype 2**	
	Assembly accession			JAUMIQ000000000		JAUMIR000000000	
	Genome sequences			GCA_030847625.1		GCA_030847615.1	
	Data Dryad Annotation accession			TBD			
Genome sequence	PacBio HiFi reads		Run	1 PACBIO_SMRT (Sequel IIe) run: 7.8 M spots, 101.2G bases, 59.6Gb			
			Accession	SRX25151365			
	Omni-C Illumina reads		Run	2 ILLUMINA (Illumina NovaSeq 6000) runs: 496.4 M spots, 149.9G bases, 49.5Gb			
			Accession	SRX25151366–7			
Genome assembly quality metrics	Assembly identifier (Quality code ^a^)				rGamWis1(8.8.P8.Q68.C99)		
	HiFi Read coverage ^b^			41.41X			
				**Haplotype 1**		**Haplotype 2**	
	Number of contigs			169		96	
	Contig N50 (bp)			141,377,706		138,191,064	
	Contig NG50 ^b^			141,377,706		138,191,064	
	Longest Contigs			293,118,657		321,148,149	
	Number of scaffolds			141		69	
	Scaffold N50			379,351,600		380,060,318	
	Scaffold NG50 ^b^			379,351,600		380,060,318	
	Largest scaffold			501,950,513		502,046,835	
	Size of final assembly			2,469,046,425		2,466,589,883	
	Phased block NG50 ^b^			141,377,706		138,191,064	
	Gaps per Gbp (# Gaps)			11(28)		11(27)	
	Indel QV (Frame shift)			48.49		48.58	
	Base pair QV			67.803		68.4573	
				Full assembly = 68.1177			
	k-mer completeness			89.2806		89.2926	
				Full assembly = 99.5467			
	BUSCO completeness (tetrapoda) *n* = 5310		**C**	**S**	**D**	**F**	**M**
		H1^c^	97.40%	96.60%	0.80%	0.80%	1.80%
		H2^c^	97.40%	96.60%	0.80%	0.90%	1.70%
	Organelles		# Partial mitochondrial sequence			JAUMIQ010000141.1	
Genome annotation quality metrics				**Count of features**			
	Genes			23,279			
	Transcripts			53,856			
	mRNA			48,567			
	lncRNA			5058			
	CDSs			48,579			
	BUSCO completeness		**C**	**S**	**D**	**F**	**M**
	(eukaryota_odb12) *n* = 129		99.20%	99.20%	0.00%	0.00%	0.80%
	(tetrapoda_odb10) *n* = 5310		98.80%	98.00%	0.80%	0.40%	0.70%
	(squamata_odb12) *n* = 11 294		98.80%	98.40%	0.40%	0.50%	0.80%

BUSCO Scores. Complete BUSCOs (C). Complete and single-copy BUSCOs (S). Complete and duplicated BUSCOs (D). Fragmented BUSCOs (F). Missing BUSCOs (M).

a Assembly quality code x.y.P.Q.C derived notation, from the study by Rhie et al. (2021), x = log10[contig NG50]; y = log10[scaffold NG50]; P = log10 [phased block NG50]; Q = Phred base accuracy QV (Quality value); C = % genome represented by the first “*n*” scaffolds, following a known karyotype for *Gambelia wislizenii* of 2*n* = 36 (Porter et al. 1994). Quality code for all the assembly denoted by primary assembly (rGamWis1.0.hap1).

b Read coverage and NGx statistics have been calculated based on the estimated genome size of 2.44 Gb.

c (H1) Haplotype 1 and (H2) haplotype 2 assembly values.

### Genome annotation

We annotated the reference assembly using the NCBI Eukaryotic Genome Annotation Pipeline (hereafter, “EGAPx”) which is published in the NCBI RefSeq database (O’Leary et al. 2016) (accessible through https://github.com/ncbi/egapx). Annotation features were identified by aligning transcripts and proteins from related taxa in the RefSeq database using BLAST (Camacho et al. 2009). Novel, species-specific RNA-Seq reads generated from seven tissue types were also aligned to the assembly using the alignment software STAR (Dobin et al. 2013). Additional features are predicted using HMM-based gene models using the NCBI Gnomon software (Souvorov et al. 2010). We evaluated the quality and completeness of our annotation by the comparing the longest protein for each annotated coding gene to eukaryotes (odb12), tetrapods (odb10), and squamates (odb12) using BUSCO (Manni et al. 2021).

## Results

### Sequencing data

**Fig. 2:**
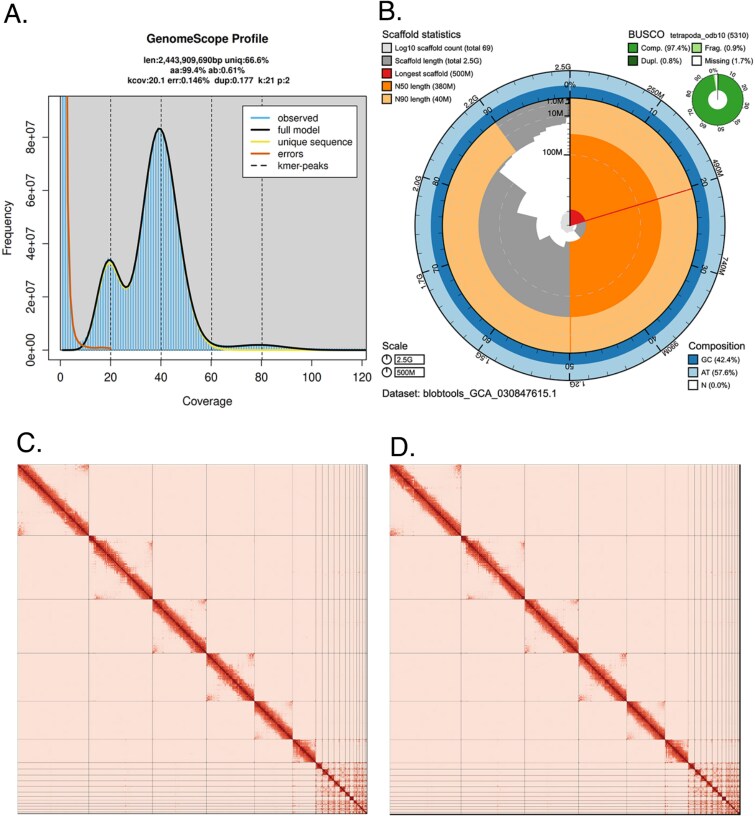
Visual overview of genome assembly metrics. A) K-mer spectra output generated from PacBio HiFi data without adapters using GenomeScope2.0. The bimodal pattern observed corresponds to a diploid genome. K-mers covered at lower coverage and low frequency correspond to differences between haplotypes, whereas the higher coverage and high-frequency k-mers correspond to the similarities between haplotypes. B) BlobToolkit Snail plot displaying the quality metrics presented in Table 2 for the *Gambelia wislizenii* primary assembly (haplotype 2; rGamWis1.0.hap2). The plot circle represents the full size of the assembly. From the inside-out, the central plot covers length-related metrics. The red line represents the size of the longest scaffold; all other scaffolds are arranged in size-order moving clockwise around the plot and drawn in gray starting from the outside of the central plot. Dark and light orange arcs show the scaffold N50 and scaffold N90 values. The central light gray spiral shows the cumulative scaffold count with a white line at each order of magnitude. White regions in this area reflect the proportion of Ns in the assembly. The dark vs. light blue area around it shows mean, maximum and minimum GS vs. AT content at 0.1% intervals (Challis et al. 2020). Omni-C contact maps for haplotype 2 C) and haplotype 1 D) genome assembly generated with PretextSnapshot. Omni-C contact maps translate proximity of genomic regions in 3D space to contiguous linear organization. Each line in the contact map corresponds to sequencing data supporting the linkage (or join) between two of such regions. Scaffolds are separated by black lines and higher density corresponds to high levels of fragmentation.

The Omni-C library generated 496.36 million read pairs and the PacBio HiFi library generated 7.75 million reads. The PacBio HiFi sequences yielded ~41X genome coverage and had an N50 read length of 13,378 bp; a minimum read length of 107 bp; a mean read length of 13,060 bp; and a maximum read length of 56,853 bp (see Supplementary Fig. S1 for read length distribution). Based on the PacBio HiFi data, Genomescope 2.0 estimated a genome size of 2.44 Gb, a 0.146% sequencing error rate, and 0.61% heterozygosity. The k-mer spectrum shows a bimodal distribution with a major coverage peak at ~ 40-fold coverage and a minor peak at ~ 20-fold coverage (Fig. 2A). Sequencing of mRNA libraries of blood, brain, intestine, liver, lung, muscle, and ovary generated 57.7, 58.1, 46.8, 67.6, 55.3, 53.3, and 55.8 M read pairs, respectively.

### Nuclear genome assembly

The final assembly (rGamWis1) consists of two pseudo haplotypes, haplotypes 1 and 2, with both genome sizes close to the estimated value from Genomescope 2.0 (Fig. 2A: Pflug et al. 2020). Haplotype 1 assembly (rGamWis1.0.hap1) consists of 141 scaffolds spanning 2.47 Gb with contig N50 of 141.37 Mb, scaffold N50 of 379.35 Mb, longest contig of 293.11 Mb, and largest scaffold of 501.95 Mb. The haplotype 2 assembly (rGamWis1.0.hap2) consists of 69 scaffolds, spanning 2.47 Gb with contig N50 of 138.19 Mb, scaffold N50 of 380.06 Mb, largest contig 321.14 Mb, and largest scaffold of 502.04 Mb. Detailed assembly statistics are reported in Table 2, and graphical representation for the haplotype 2 assembly is shown in Fig. 2B (see Supplementary Fig. S1 for the haplotype 1 assembly).

The haplotype 1 assembly has a BUSCO completeness score of 97.4% using the Tetrapoda gene set, a per-base quality (QV) of 67.8, a k-mer completeness of 89.28% and a frameshift indel QV of 48.49; while the haplotype 2 assembly has a BUSCO completeness score of 97.4% using the same gene set, a per-base quality (QV) of 68.45, a k-mer completeness of 89.29%, and a frameshift indel QV of 48.58.

During manual curation, we generated 1 break and 11 joins on haplotype 1 and 2 breaks and 15 joins on haplotype 2. We were able to close two gaps on haplotype 2. No further contigs were modified or removed. The Omni-C contact maps show highly contiguous assemblies with chromosome-length scaffolds (Fig. 2B). The six largest scaffolds range from 163.1 to 502.1 Mb, the next 12 largest scaffolds range from 11.7 to 45.5 Mb, and all remaining scaffolds range from 10.8 to 37.5 Kb. Assembly statistics are reported in Table 2 and represented graphically in Fig. 2C. Both assemblies are now deposited on NCBI (see Table 2 and Data availability for details).

We assembled a partial mitochondrial genome from the PacBio HiFi reads using the reference-guided pipeline MitoHiFi. The final mitochondrial sequence has a size of 25,077 bp, with a base composition of A = 29.72%, C = 21.27%, G = 10.38%, and T = 29.14%, with 13 unique transfer RNAs.

Our final genome annotation included 23,279 genes, with a tetrapod BUSCO completeness of 98.9%. A list of annotation statistics and BUSCO score breakdowns for eukaryotes, tetrapods, and squamates is reported in Table 2.

## Discussion

Here we present a high-quality reference genome for *G. wislizenii*. The excellent quality of the genome is exemplified by BUSCO completeness scores of 97.4% for both haplotypes, and on a scaffold distribution that is consistent with the known chromosome arrangement for the species. The 2*n* = 36 karyotype of *G. wislizenii* is composed of six pairs of macrochromosomes and 12 pairs of microchromosomes (Porter et al. 1994). Haplotype 2 of the *G. wislizenii* genome presented here includes six scaffolds that are consistent in size with macrochrosomes (163.1 to 502.1 Mb), 12 additional scaffolds that are consistent in size with microchromsomes (11.7 to 45.5 Mb), and a remaining set of 51 scaffolds that are much smaller (10.8 to 37.5 Kb) and most likely comprise difficult-to-assemble sequences rich in repeats.

There are now 217 reference genomes representing squamate reptiles uploaded onto NCBI. Of these, 40 represent the major lizard clade, Iguania, a cosmopolitan assemblage with nearly 2,100 currently recognized species (Uetz et al. 2024). Iguania is divided into 14 families, one of which is Crotaphytidae, and the *G. wislizenii* genome reported here is the first for this small but deeply divergent iguanian clade composed of just two genera and 12 species. More than half of the available iguanian genomes (*n* = 23) are concentrated among the Old World acrodontan families Agamidae (*n* = 13) and Chameleonidae (*n* = 10). Among the 12 families of pleurodont iguanians, only five of 12 families currently have a reference genome posted on NCBI: Anolidae (*n* = 5), Corytophanidae (*n* = 1), Iguanidae (*n* = 3), Phrynosomatidae (*n* = 7), and now Crotaphytidae (*n* = 1). The genome presented here will thus provide a valuable resource for broader comparative genomic studies spanning the hyper-diverse Iguania, as well as for studies investigating the conservation, ecology, and evolutionary history of *G. wislizenii*, and its close relatives within the genera *Gambelia* and *Crotaphytus*.

From a conservation perspective, we note that *Gambelia* includes one of the most highly endangered reptiles in the United States, *G. sila*, as well as one that barely enters the country at its southern border, *G. copei*. Having access to genomic resources across a dense sample spanning all three species will be critically important for assessing the conservation genetics, population demography, and long-term viability of these species in California. As a captive breeding and release program is already underway for *G. sila*, having both this reference genome and the landscape-level analysis of the species’ genomic variation will help inform breeding strategies, while maintaining the naturally occurring genomic variation that characterizes the species. These landscape genomic resources to be generated via the CCGP will also offer the potential to investigate the genetic underpinnings of important features of the biology of leopard lizards, including gravid and breeding coloration, territoriality, and reversals in patterns of sexual dimorphism, as well as the extent and nature of gene flow at *Gambelia* species boundaries. Understanding historical gene flow among the species will, more broadly, also shed light on landscape scale processes, such as the primordial separation of the San Joaquin and Mojave Deserts and their biotas, which in turn can inform conservation practices for many species in those biomes.

## Data Availability

Data generated for the assembly in this study are available under NCBI BioProject PRJNA986198. Raw sequencing data for sample HBS135999 (NCBI BioSample SAMN35845454) are deposited in the NCBI Short Read Archive (SRA) under SRR29647190 for PacBio HiFi sequencing data, and SRR29647188, SRR29647189 for the Omni-C Illumina sequencing data. GenBank accessions for both the haplotype 1 and haplotype 2 assemblies are GCA_030847625.1 and GCA_030847615.1; and for genome sequences JAUMIQ000000000 and JAUMIR000000000. The GenBank genome assembly for the mitochondrial genome is JAUMIQ010000141.1. Raw RNA sequencing data for MVZ:Herp:300824 (as HBS135999) generated for the annotation (NCBI BioSamples SAMN40863703, SAMN40863704, SAMN40863705, SAMN40863706, SAMN40863707, SAMN40863708, and SAMN40863709), are deposited in the NCBI SRA (SRR29347968, SRR29347967, SRR29347966, SRR29347965, SRR29347964, SRR29347963, and SRR29347962, respectively), and are available under NCBI BioProject PRJNA1011925. The annotation will be made available with the assembly GCA_030847625.1. Assembly scripts and other data for the analyses presented can be found at the following GitHub repository: www.github.com/ccgproject/ccgp_assembly.

## References

[ref1] Abdennur N, Mirny LA. Cooler: scalable storage for Hi-C data and other genomically labeled arrays. Bioinformatics. 2020;36:311–316. 10.1093/bioinformatics/btz540 31290943 PMC8205516

[ref2] Allio R, Schomaker-Bastos A, Romiguier J, Prosdocimi F, Nabholz B, Delsuc F. MitoFinder: efficient automated large-scale extraction of mitogenomic data in target enrichment phylogenomics. Mol Ecol Resour. 2020;20:892–905. 10.1111/1755-0998.13160 32243090 PMC7497042

[ref3] Beninde J, Toffelmier E, Shaffer HB. A brief history of population genetic research in California and an evaluation of its utility for conservation decision-making. J Hered. 2022;113:604–614. 10.1093/jhered/esac049 36056714 PMC9709982

[ref4] Bishop AP, Westeen EP, Yuan ML, Escalona M, Beraut E, Fairbairn C, Marimuthu MPA, Nguyen O, Chumchim N, Toffelmier E, et al. Assembly of the largest squamate reference genome to date: the western fence lizard. J Hered. 2023;114:521–528. 10.1093/jhered/esad037 37335574 PMC10445515

[ref5] Camacho C, Coulouris G, Avagyan V, Ma N, Papadopoulos J, Bealer K, Madden TL. BLAST+: architecture and applications. BMC Bioinformatics. 2009;10:421–421. 10.1186/1471-2105-10-421 20003500 PMC2803857

[ref6] Challis R, Richards E, Rajan J, Cochrane G, Blaxter M. BlobToolKit—interactive quality assessment of genome assemblies. G3 (Bethesda). 2020;10:1361–1374. 10.1534/g3.119.400908 32071071 PMC7144090

[ref1c] Challis R, Kumar S, Sotero-Caio C, Brown M, Blaxter M. Genomes on a Tree (GoaT): A versatile, scalable search engine for genomic and sequencing project metadata across the eukaryotic tree of life. Wellcome Open Res. 2023;8:24. 10.12688/wellcomeopenres.18658.1 36864925 PMC9971660

[ref7] Cheng H, Jarvis ED, Fedrigo O, Koepfli K-P, Urban L, Gemmell NJ, Li H. Haplotype-resolved assembly of diploid genomes without parental data. Nat Biotechnol. 2022:40:1332–1335. 10.1038/s41587-022-01261-x PMC9464699 35332338

[ref8] Dobin A, Davis CA, Schlesinger F, Drenkow J, Zaleski C, Jha S, Batut P, Chaisson M, Gingeras TR. STAR: ultrafast universal RNA-seq aligner. Bioinformatics. 2013;29:15–21. 10.1093/bioinformatics/bts635 23104886 PMC3530905

[ref9] Fiedler PL, Erickson B, Esgro M, Gold M, Hull JM, Norris J, Shapiro B, Westphal M, Toffelmier E, Shaffer HB. Seizing the moment: the opportunity and relevance of the California Conservation Genomics Project to state and federal conservation policy. J Hered. 2022;113:589–596. 10.1093/jhered/esac046 36136001 PMC9709969

[ref10] Germano DJ, Williams DF, Tordoff W III. Effect of drought on blunt-nosed leopard lizards (*Gambelia sila*). Northwest Nat. 1994;75:11–19. 10.2307/3536555

[ref11] Germano DJ, Smith PT, Tabor SP. Food habits of the blunt-nosed leopard lizard (*Gambelia sila*). Southwest Nat. 2007;52:318–323. 10.1894/0038-4909(2007)52[318:FHOTBL]2.0.CO;2

[ref12] Germano DJ, Rathbun GB, Saslaw LR, Cypher BL, Cypher EA, Vredenburgh LM. The San Joaquin Desert of California: ecologically misunderstood and overlooked. Nat Areas J. 2011;31:138–147. 10.3375/043.031.0206

[ref13] Ghurye J, Pop M, Koren S, Bickhart D, Chin C-S. Scaffolding of long read assemblies using long range contact information. BMC Genomics. 2017;18:527. 10.1186/s12864-017-3879-z 28701198 PMC5508778

[ref14] Ghurye J, Rhie A, Walenz BP, Schmitt A, Selvaraj S, Pop M, Koren S. Integrating Hi-C links with assembly graphs for chromosome-scale assembly. PLoS Comp Biol. 2019;15:e1007273. 10.1371/journal.pcbi.1007273 PMC6719893 31433799

[ref16] Gurevich A, Saveliev V, Vyahhi N, Tesler G. QUAST: quality assessment tool for genome assemblies. Bioinformatics. 2013;29:1072–1075. 10.1093/bioinformatics/btt086 23422339 PMC3624806

[ref17] Hansen RW, Shedd JD. California amphibians and reptiles. Princeton (NJ, USA): Princeton University Press; 2025. 10.2307/jj.17453116

[ref18] Kerpedjiev P, Abdennur N, Lekschas F, McCallum C, Dinkla K, Strobelt H, Gehlenborg N. HiGlass: web-based visual exploration and analysis of genome interaction maps. Genome Biol. 2018;19:125. 10.1186/s13059-018-1486-1 30143029 PMC6109259

[ref19] Korlach J, Gedman G, Kingan SB, Chin C-S, Howard JT, Audet J-N, Cantin L, Jarvis ED, Jarvis ED. De novo PacBio long-read and phased avian genome assemblies correct and add to reference genes generated with intermediate and short reads. GigaScience. 2017;6:1–16. 10.1093/gigascience/gix085 PMC5632298 29020750

[ref21] Lappin AK, Swinney EJ. Sexual dimorphism as it relates to natural history of leopard lizards (Crotaphytidae: *Gambelia*). Copeia. 1999;1999:649. 10.2307/1447597

[ref22] Lee S, Bakker CR, Vitzthum C, Alver BH, Park PJ. Pairs and Pairix: a file format and a tool for efficient storage and retrieval for Hi-C read pairs. Bioinformatics. 2022;38:1729–1731. 10.1093/bioinformatics/btab870 34978573 PMC10060703

[ref23] Li H . Aligning sequence reads, clone sequences and assembly contigs with BWA-MEM. arXiv. 2013, preprint: not peer reviewed. Retrieved from http://arxiv.org/abs/1303.3997

[ref24] Mahrdt CR, McGuire JA, Beaman KR. *Gambelia copeii* (Yarrow) Cope’s leopard lizard. Cat Am Amphib Reptiles. 2010;871:1–8.

[ref25] Manni M, Berkeley MR, Seppey M, Simao FA, Zdobnov EM. BUSCO update: novel and streamlined workflows along with broader and deeper phylogenetic coverage for scoring of eukaryotic, prokaryotic, and viral genomes. Mol Biol Evol. 2021;38:4647–4654. 10.1093/molbev/msab199 34320186 PMC8476166

[ref26] McCoy CJ . Natural history notes on *Crotaphytus wislizeni* (Reptilia: Iguanidae) in Colorado. Amer Midl Nat. 1967;77:138–146. 10.2307/2423433

[ref27] McGuire JA . Phylogenetic systematics of crotaphytid lizards (Reptilia: Iguania: Crotaphytidae). Bull Carnegie Mus Nat Hist. 1996;32:1–143. 10.5962/p.240775

[ref28] Montanucci RR . Observations on the San Joaquin leopard lizard, *Crotaphytus wislizenii silus* Stejneger. Herpetologica. 1965;21:270–283.

[ref29] Montanucci RR . Analysis of hybridization between *Crotaphytus wisizenii* and *Crotaphytus silus* (Sauria: Iguanidae) in California. Copeia. 1970;1970:104. 10.2307/1441979

[ref30] O’Leary NA, Wright MW, Brister JR, Ciufo S, Haddad D, McVeigh R, Rajput B, Robbertse B, Smith-White B, Ako-Adjei D, et al. Reference sequence (RefSeq) database at NCBI: current status, taxonomic expansion, and functional annotation. Nucleic Acids Res. 2016;44:D733–D745. 26553804 10.1093/nar/gkv1189 PMC4702849

[ref31] Open2C, Abdennur N, Fudenberg G, Flyamer IM, Galitsyna AA, Goloborodko A, Imakaev M, Venev SV. Pairtools: from sequencing data to chromosome contacts. PLOS Comp Biol. 2024;20:e1012164. 10.1371/journal.pcbi.1012164 PMC11164360 38809952

[ref32] Parker WS, Pianka ER. Ecological observations on the leopard lizard (*Crotaphytus wislizeni*) in different parts of its range. Herpetologica. 1976;1:95–114.

[ref33] Pflug JM, Holmes VR, Burrus C, Johnston JS, Maddison DR. Measuring genome sizes using read-depth, k-mers, and flow cytometry: methodological comparisons in beetles (Coleoptera). G3 (Bethesda). 2020;10:3047–3060. 10.1534/g3.120.401028 32601059 PMC7466995

[ref34] Porter CA, Haiduk MW, de Queiroz K. Evolution and phylogenetic significance of ribosomal gene location in chromosomes of squamate reptiles. Copeia. 1994;1994:302. 10.2307/1446980

[ref35] Ramírez F, Bhardwaj V, Arrigoni L, Lam KC, Grüning BA, Villaveces J, Manke T. High-resolution TADs reveal DNA sequences underlying genome organization in flies. Nat Commun. 2018;9:189. 10.1038/s41467-017-02525-w 29335486 PMC5768762

[ref36] Ranallo-Benavidez TR, Jaron KS, Schatz MC. GenomeScope 2.0 and Smudgeplot for reference-free profiling of polyploid genomes. Nat Commun. 2020;11:1432. 32188846 10.1038/s41467-020-14998-3 PMC7080791

[ref37] Rhie A, Walenz BP, Koren S, Phillippy AM. Merqury: reference-free quality, completeness, and phasing assessment for genome assemblies. Genome Biol. 2020;21:245. 10.1186/s13059-020-02134-9 32928274 PMC7488777

[ref38] Rhie A, McCarthy SA, Fedrigo O, Damas J, Formenti G, Koren S, Uliano-Silva M, Chow W, Fungtammasan A, Kim J, et al. Towards complete and error-free genome assemblies of all vertebrate species. Nature. 2021;592:737–746. 10.1038/s41586-021-03451-0 33911273 PMC8081667

[ref39] Richmond JQ, Wood DA, Westphal MF, Vandergast AG, Leaché AD, Saslaw LR, Butterfield HS, Fisher RN. Persistence of historical population structure in an endangered species despite near-complete biome conversion in California’s San Joaquin Desert. Mol Ecol. 2017;26:3618–3635. 10.1111/mec.14125 28370723

[ref40] Richmond JQ, McGuire JA, Escalona M, Marimuthu MPA, Nguyen O, Sacco S, Beraut E, Toffelmier E, Fisher RN, Wang IJ, et al. Reference genome of an iconic lizard in western North America, Blainville’s horned lizard *Phrynosoma blainvillii*. J Hered. 2023;114:410–417. 10.1093/jhered/esad032 37195437

[ref41] Shaffer HB, Toffelmier E, Corbett-Detig RB, Escalona M, Erickson B, Fiedler P, Gold M, Harrigan RJ, Hodges S, Luckau TK, et al. Landscape genomics to enable conservation actions: the California Conservation Genomics Project. J Hered. 2022;113:577–588. 10.1093/jhered/esac020 35395669

[ref42] Sim SB, Corpuz RL, Simmonds TJ, Geib SM. HiFiAdapterFilt, a memory efficient read processing pipeline, prevents occurrence of adapter sequence in PacBio HiFi reads and their negative impacts on genome assembly. BMC Genomics. 2022;23:157. 10.1186/s12864-022-08375-1 35193521 PMC8864876

[ref43] Souvorov A, Kapustin Y, Kiryutin B, Chetvernin V, Tatusova T, Lipman D. Gnomon—NCBI eukaryotic gene prediction tool. Bethesda, MD: National Center for Biotechnology Information; 2010:1–24.

[ref44] Stewart JAE, Butterfield HS, Richmond JQ, Germano DJ, Westphal MF, Tennant EN, Sinervo B. Habitat restoration opportunities, climate niche contraction, and conservation biogeography in California’s San Joaquin Desert. PlosOne. 2019;14:e0210766. 10.1371/journal.pone.0210766 PMC6333358 30645624

[ref45] Tanner WW, Krogh JE. Ecology of the leopard lizard, *Crotaphytus wislizeni* at the Nevada test site, Nye County. Nevada Herpetologica. 1974;30:63–72.

[ref46] Toffelmier E, Beninde J, Shaffer HB. The phylogeny of California, and how it informs setting multi-species conservation priorities. J Hered. 2022;113:597–603. 10.1093/jhered/esac045 36048626 PMC9709974

[ref47] Tollestrup K . The social behavior of two species of closely related leopard lizards, *Gambelia silus* and *Gambelia wislizenii*. Z Tierpsychol. 1983;62:307–320. 10.1111/j.1439-0310.1983.tb02159.x

[ref48] Uetz P, Freed P, Aguilar R, Reyes F, Kudera J, Hošek J, editors. The Reptile Database; 2024. accessed 2024 December 27. http://www.reptile-database.org

[ref49] Uliano-Silva M, Ferreira JGRN, Krasheninnikova K, Darwin Tree of Life Consortium, Blaxter M, Mieszkowska N, Hall N, Holland P, Durbin R, Richards T, et al. MitoHiFi: a python pipeline for mitochondrial genome assembly from PacBio high fidelity reads. BMC Bioinformatics. 2023;24:288. 10.1186/s12859-023-05385-y 37464285 PMC10354987

